# Understanding the Community Perceptions and Knowledge of Bats and Transmission of Nipah Virus in Bangladesh

**DOI:** 10.3390/ani10101814

**Published:** 2020-10-05

**Authors:** Mohammad Mahmudul Hassan, Md. Abul Kalam, Mahabub Alam, Shahanaj Shano, Abdullah Al Faruq, Md. Saddam Hossain, Md. Nurul Islam, Shahneaz Ali Khan, Ariful Islam

**Affiliations:** 1 Faculty of Veterinary Medicine, Chattogram Veterinary and Animal Sciences University, Chattogram 4225, Bangladesh; mahabub38@yahoo.com (M.A.); faruqabdullahal103@gmail.com (A.A.F.); drsaddamcvasu@gmail.com (M.S.H.); shahneazbat@gmail.com (S.A.K.); 2 Helen Keller International, Dhaka 1212, Bangladesh; a.kalam724@gmail.com; 3 Institute of Epidemiology, Disease Control and Research (IEDCR), Dhaka 1212, Bangladesh; shahanajshano@gmail.com; 4 Global Health Development (GHD) and EMPHNET, Amman 11195, Jordan; nurul.dvm@gmail.com; 5 Centre for Integrative Ecology, School of Life and Environmental Science, Deakin University, Geelong Campus, VIC 3216, Australia; 6 EcoHealth Alliance, New York, NY 10001-2320, USA

**Keywords:** bat ecology, community perception, conservation, myth, Nipah virus, Bangladesh

## Abstract

**Simple Summary:**

We assessed people’s knowledge, attitudes, and perceptions regarding bat ecology, myths associated with bats, and their involvement in the transmission of Nipah virus (NiV). We found that community people in Bangladesh had inadequate knowledge of bat ecology and myths surrounding NiV. People’s demographic characteristics, such as sex, age, occupation, level of education, and exposure to a Nipah outbreak, were determined to be key factors influencing their knowledge, attitudes, and perceptions of bat ecology, myths, and their transmission of NiV. Educational interventions are recommended for targeted groups in the community to raise awareness and to improve people’s current knowledge of the role of bats in ecosystem services and their risky behavioral practices driving NiV transmission in Bangladesh.

**Abstract:**

Bats are known reservoirs of Nipah virus (NiV) and some filoviruses and also appear likely to harbor the evolutionary progenitors of severe acute respiratory syndrome coronavirus (SARS-CoV), severe acute respiratory syndrome coronavirus-2 (SARS-CoV-2), and Middle East respiratory syndrome coronavirus (MERS-CoV). While bats are considered a reservoir of deadly viruses, little is known about people’s knowledge, attitudes, and perceptions of bat conservation and ecology. The current study aimed to assess community people’s knowledge, attitudes, and perceptions of bat ecology, myths, and the role of bats in transmitting NiV in Bangladesh. Since 2001, NiV has been a continuous threat to public health with a mortality rate of approximately 70% in Bangladesh. Over the years, many public health interventions have been implemented to raise awareness about bats and the spreading of NiV among the community peoples of Nipah outbreak areas (NOAs) and Nipah non-outbreak areas (NNOAs). We hypothesized that people from both areas might have similar knowledge of bat ecology and myths about bats but different knowledge regarding their role in the spreading of NiV. Using a four-point Likert scale-based questionnaire, our analysis showed that most people lack adequate knowledge regarding the role of bats in maintaining the ecological balance and instead trust their beliefs in different myths about bats. Factor score analysis showed that respondents’ gender (*p* = 0.01), the outbreak status of the area (*p* = 0.03), and their occupation (*p* = 0.04) were significant factors influencing their knowledge of bat ecology and myths. A regression analysis showed that farmers had 0.34 times the odds of having correct or positive knowledge of bat ecology and myths than businesspersons (odds ratio (OR) = 0.34, 95% confidence interval (95% CI) = 0.15–0.78, *p* = 0.01). Regarding the spreading of NiV via bats, people had a lower level of knowledge. In NOAs, age (*p* = 0.00), occupation (*p* = 0.00), and level of education (*p* = 0.00) were found to be factors contributing to the amount of knowledge regarding the transmission of NiV, whereas in NNOAs, the contributing factors were occupation (*p* = 0.00) and level of education (*p* = 0.01). Regression analysis revealed that respondents who were engaged in services (OR = 3.02, 95% CI = 1.07–8.54, *p* = 0.04) and who had completed primary education (OR = 3.06, 95% CI = 1.02–9.17, *p* < 0.05) were likely to have correct knowledge regarding the spreading of NiV. Based on the study results, we recommend educational interventions for targeted groups in the community, highlighting the ecosystem services and conservation of bats so as to improve people’s current knowledge and subsequent behavior regarding the role of bats in ecology and the spreading of NiV in Bangladesh.

## 1. Introduction

Bats are considered among the most ignored and mysterious mammals worldwide, and with 1300 bat species found across the globe, they constitute the second largest group of mammals on this planet [1]. From a global ecology perspective, they play an enormous role in pollination, seed dispersal, and pest control [2]. The existence of bats is observed in all habitats, from high mountains to deserts, and currently, many species are threatened or endangered [3]. Bats play a significant role in ecosystems and act as important bio-indicators, providing vital ecosystem services valued by humans [4]. The ecological significance of bats is widely recognized, notably in those areas where there are various symbioses between bats and plants that are necessary for their survival [5,6]. However, little is known about how people perceive bats and their role in ecology and conservation. Using a questionnaire survey, Lim and Wilson found that having tertiary education and knowledge of the role of bats in ecology resulted in a positive attitude regarding the conservation of bats [7]. In another study, it was revealed that older and more educated people had a more positive attitude toward bats [8]. Addressing local people’s traditional knowledge is critical in designing an active surveillance system to conserve any species [9]. Like with other wildlife, it is important to learn community people’s knowledge, attitudes, and perceptions of bats. Most importantly, community people’s active participation in decision- and policy-making procedures in the conservation process, as well as their knowledge and attitudes, can bring about better outcomes for the conservation of most species [10]. Moreover, better understanding, familiarity, experience, desire to protect wildlife, and perceptions of wildlife conservation can improve local community people’s attitudes concerning the conservation of species [11,12]. In one study, it was found that knowledge and awareness of wildlife may vary based on people’s demographic and socioeconomic characteristics, such as age, gender, level of education, level of income, and living closer to wildlife, as these determinants act as behavior modifiers [13]. More knowledge and an advanced level of education appear to have a positive impact on people’s attitudes toward animals as well as the associated conservation efforts [14].

Bats can carry viruses that are deadly to humans, including Ebola virus, Marburg virus, SARS-CoV, rabies virus, and Nipah virus (NiV) [15]. During the Nipah encephalitis outbreak in Malaysia during 1999, pigs acted as an intermediate host between bats and humans [16,17]. NiV can also be transmitted from bats to humans without an intermediate host [18], for example, through consumption of fresh date palm sap contaminated with bat saliva or urine in Bangladesh [19], as bats visit and contaminate the date palm sap regularly during the night [20]. Subsequent communication of NiV to humans following person-to-person transmission of the virus is fatal [21]. There are some useful measures and tools to prevent NiV infection, such as boiling date palm sap prior to drinking, use of a bamboo skirt (bat shield) to avoid bats contacting the date palm sap, and processing of the date palm sap to the product “gur” (a boiled and processed product). In addition, bat–human interactions—particularly bat roosts near human dwellings—exposure to bats, and bat hunting may increase the risk of Nipah infection in Bangladesh [22]. However, previous studies on community people’s knowledge, perceptions, and attitudes of bat ecology and conservation are lacking in Bangladeshi settings. Therefore, this is the first research study in Bangladesh where people’s knowledge, perceptions, and attitudes were assessed regarding bat ecology and myths pertaining to bats. For the past few years, people have gained knowledge from media coverage or health community workers about NiV and the origin of this infection through bats. There are 33 species of bats found in Bangladesh, of which fruit bats of the *Pteropus* genus are natural hosts for NiV transmission [23]. The loss of cultivated fruits by farmers because of fruit bats has caused local people to develop more negative attitudes toward bats, based on incorrect information, in some areas of the world [23,24], which has resulted in a lack of understanding of bats by community people.

Nevertheless, due to the lack of knowledge and awareness, community people cannot take advantage of methods to mitigate Nipah infection as detailed above, resulting in periodic outbreaks of NiV infections among community people in some areas of Bangladesh [25]. One of the common corridors for transmitting NiV to humans is the consumption of urine-contaminated tari (a traditional liquor made from raw date palm sap) or raw date palm sap [26]. The greatest risk factors for human NiV infection in a particular area are the size of the bat population, the number of date palm trees used for juice production, and human consumption of raw date palm juice [27]. People’s activities and attitudes can influence bat conservation; hence, before starting an education and awareness program, a baseline survey is essential to understand the strength of knowledge already prevailing among community people. When an NiV outbreak occurs, even if only a single NiV case is identified, public health and community workers alert the local community in the Nipah outbreak areas (NOAs) regarding this NiV transmission. Usually, these educational awareness campaigns do not focus on bat ecology, myths about bats, and the role of bats in conservation. As such, we hypothesized that the depth of knowledge, attitudes, and perceptions of bat ecology and myths about bats might be similar between NOAs and NNOAs but that there might be differences in knowledge on the spreading of NiV. Therefore, this study aimed to assess people’s knowledge, attitudes, and perceptions regarding bat ecology, public myths about bats, and the transmission dynamics of NiV in Bangladesh.

## 2. Materials and Methods

### 2.1. Study Areas

**Figure 1:**
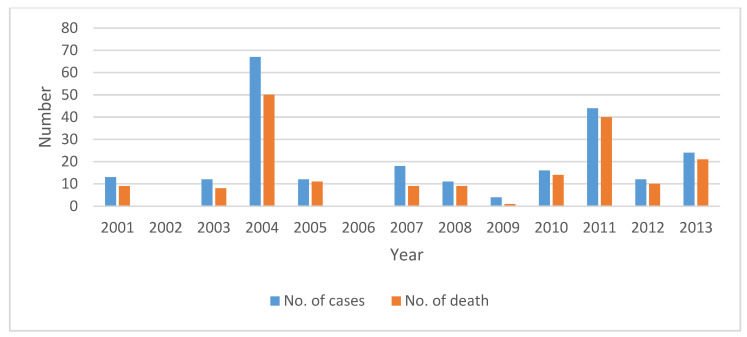
Number of cases and deaths due to human Nipah virus (NiV) infections in Bangladesh, 2001–2013.

**Figure 2:**
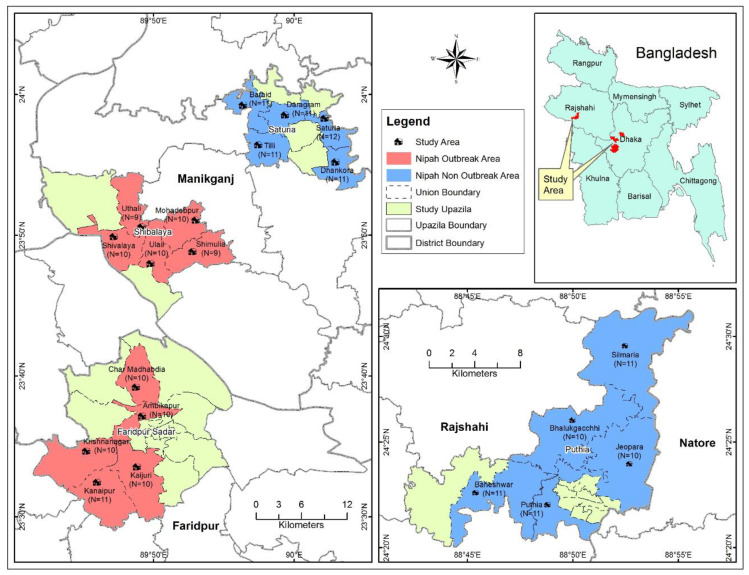
The spatial distribution of the study sites with the number of respondents (*n*) in Nipah outbreak areas (NOAs; depicted in pink) and Nipah non-outbreak areas (NNOAs; depicted in light green). The map was plotted using ArcMap, version 10.2, Environmental Systems Research Institute, Redlands, CA, USA.

We conducted this study in two NOAs and two NNOAs selected from three districts of Bangladesh, namely, Manikganj (one outbreak area (Shivalya Upazila) and one non-outbreak area (Saturia Upazila)), Faridpur (one outbreak area (Faridpur Sadar Upazila)), and Rajshahi (one non-outbreak area (Puthia Upazila)). Upazila (a subdistrict) is the lowest administrative boundary of an area in Bangladesh. The subdistricts that identified or reported NiV cases were considered NOAs, while those that did not were considered NNOAs. NiV infection data between 2001 and 2013, retrieved from the World Health Organization website [28], and the outbreak situation of NiV infections in Bangladesh were analyzed and the results are provided in Figure 1. The selected NOAs are neighbors to the NNOAs (Figure 2). The NOA study sites were selected based on the higher density of date palm trees, the availability of bat roosting sites, and the number of NiV outbreaks, while NNOAs were selected based on the comparatively lower density of date palm trees, the lower number of bat roosting sites, and no NiV outbreaks (dataset is available from the corresponding author on request). A higher date palm tree density and a greater number of roosting sites are considered to facilitate higher risk of exposure to bats and NiV transmission of NOAs compared to NNOAs [27,29,30]. By contrast, community people from NNOAs are less likely to interact with bats, which may be reflected in their level of knowledge on bats and NiV transmission.

### 2.2. Data Collection

We conducted a survey from January to February 2014 following an NiV outbreak in Bangladesh with several human deaths in 2013 [28]. We aimed to assess the community knowledge of bat ecology, the myths about bats, and the perceptions regarding the spreading of NiV through bats. Initially, using power analysis (STATA/IC-13), we determined that the minimum number of interviews with community people (5 villages from each Upazila and 10 respondents from each village) that needed to be conducted for our study was 50. Using STATA/IC-13 software, we determined that a sample size of 50 would be necessary to detect an expected effect size of Z (1.96) at an alpha level of 5% and power of 90%. However, the ultimate sample size varied slightly due to some unavoidable circumstances. Finally, we collected information from 99 community interviewees from NOAs (Shivalya Upazila, *n* = 48; Faridpur Sadar Upazila, *n* = 51) and 109 from NNOAs (Saturia Upazila, *n* = 56; Puthia Upazila, *n* = 53) (Table 1) using a pre-prepared and pretested paper-based questionnaire. A slight modification to ensure the suitability of language was made based on pretesting. We obtained verbal and written consent from each respondent/participant during the interview. We collected data on five different categories of closed-ended questions, including bat behavior, morphology, diversity, and importance related to the knowledge of bat ecology; available myths on bats; and perceptions of their role in spreading NiV. During the analysis phase, these categories were re-coded into two main themes: (1) bat ecology and myths about bats, and (2) the spreading of NiV. We measured the responses on a four-point Likert scale (4 = strongly agree (SA), 3 = agree (A), 2 = disagree (DA), 1 = strongly disagree (SD)), where the items were either positive or negative. The negative items were reversed during the data analysis phase and calculated accordingly.

### 2.3. Data Analysis

We performed data analysis using statistical analytical tool STATA/IC-13.1 (StataCorp, 4905, Lake Way Drive, College Station, TX 77845, USA). Cronbach’s alpha was used to measure the internal consistency of the questionnaire. At the same time, the reproducibility was evaluated using intra-class correlations for each item regarding the knowledge, perceptions, and attitudes of ecology and myths about bats and the spreading of NiV, with an acceptable value being ≥0.69. The calculation for Cronbach’s alpha was set as 0.68 for bat ecology and myths and 0.42 for the spreading of NiV, which is not a satisfactory value [31]. However, as knowledge on NiV transmission is an important thematic area in our study design, we performed a set of tests (principle factor method and chi-square) to determine the significance of the items. To analyze the data, we used descriptive statistics, such as frequencies and percentages. However, to facilitate a comparison, the responses on the spreading of NiV were analyzed by NNOAs and NOAs. Relationships between independent samples were explored using the chi-square test to determine if there were differences among respondents’ characteristics concerning the themes. Using the principal factor method [32], we identified significant factors in terms of demographic characteristics and themes. Outcomes regarding bat ecology and myths about bats in addition to perceptions of the spreading of NiV were categorized as “negative” versus “positive” and “incorrect” versus “correct,” respectively. Furthermore, this factor score analysis was also used as part of multivariate logistic regression analysis to determine the association with key themes regarding respondents’ demographics. Results are expressed as odds ratios (ORs) accompanied by 95% confidence intervals (95% CIs), and *p*-value < 0.05 was used as the threshold for statistical significance. 

### 2.4. Ethical Statement

All subjects gave their informed consent for inclusion before they participated in the study. The study was conducted in accordance with the Declaration of Helsinki, and the protocol was approved by the Ethics Committee of the Chattogram Veterinary and Animal Sciences University, Bangladesh (permit ref. no. CVASU/Dir (R and E) AEEC/2015/02).

## 3. Results

### 3.1. Demographic Characteristics 

We conducted a total of 208 interviews for this study (survey data are available in Table S1). The characteristics of the study interviewees are shown in Table 1. Out of the 208 interviews, 109 were carried out in NNOAs, while the other 99 interviews took place in NOAs. Most of the participants were male (*n* = 184) and belonged to the 25–44 age group (*n* = 139). In terms of occupation, most of the participants were farmers (*n* = 89), while close to half of the participants had completed the primary level of education (*n* = 97).

### 3.2. Knowledge, Attitudes, and Perceptions of Bat Ecology and Myths 

We used the responses based on 16 Likert scale items to understand participants’ knowledge and perceptions of bat ecology and myths about bats (Table 2). Most respondents (*n* = 169) disagreed (SD + DA) with the statement “Bats are birds”, demonstrating correct knowledge that bats are not birds. Regarding the item “Bats are dirty animals”, mixed perceptions were recorded from the responses. Most of the respondents (*n* = 132) agreed (SA + A) with this statement, reflecting an overall negative perception toward bats. Similarly, a little over half of the respondents (*n* = 112) agreed (SA + A) that “Bats are blind”, representing incorrect knowledge of bats’ eyesight. On the contrary, while asking about whether “Bats lay eggs”, more than two-thirds of the respondents (*n* = 166) disagreed (D + SD) with this statement, which reflects respondents’ correct knowledge of bats’ reproductive characteristics. On the other hand, most of the participants (*n* = 137) disagreed (D + SD) with the statement that “Bats look like foxes”. 

While asking whether “Bats eat and defecate in the same place (i.e., the mouth)”, most of the respondents (*n* = 149) agreed (SA + A) with this item, reflecting fairly incorrect knowledge of the anatomy of bats. A similar pattern was observed while asking whether “Bat meat and bones can cure different diseases”. More than half of the respondents (*n* = 107) agreed (SA + A) with this statement, while the others (*n* = 101) disagreed (SD + D). The results on “Bats can get entangled in hair” shows an interesting observation. More than half of the respondents (*n* = 112) disagreed (SD + D) with this statement. While asking whether “Bats are important in nature”, most of the respondents (*n* = 137) agreed with this statement. However, the results of the statement “I am not interested in whether bats in Bangladesh are endangered”, indicating that respondents were reluctant for bats to disappear. More than half of the respondents agreed (SA + A) with this statement (*n* = 130). Interestingly, the majority of the respondents (*n* = 153) agreed (SA + A) that “Bat populations are decreasing day by day”, which was a little less than of those who disagreed and strongly disagreed (*n* = 153 and *n* = 55, respectively). An interesting finding was observed while asking whether “Greater attention should be provided for bat protection”. Agreement (SA + A) and disagreement (SD + D) was almost split in half with this statement (*n* = 106 and *n* = 102, respectively).

On the other hand, most of the respondents (*n* = 154) disagreed (SD + D) with the statement “Bats always attack human eyes”. Similarly, the statement “All bats suck blood from humans” demonstrated that the majority of the respondents (*n* = 170) disagreed.

While asking whether “Bats are a sign of bad things”, the analysis showed mixed perceptions. A little over half of the respondents (*n* = 117) disagreed (SD + D) with this statement, while less than half (*n* = 91) of the respondents agreed (SA + A). On the other hand, the results regarding “I like to read books about bats” demonstrated that most of the respondents disagreed (SD + D) with this statement, indicating that they were not interested in exploring about bats.

#### Differences in Respondents’ Knowledge, Perceptions, and Attitudes on Bat Ecology and Myths 

Principle factor analysis was performed to show the significant (*p*-values < 0.05) factors between the demographic variables and the theme of bat ecology and myths. The results are demonstrated in Table 3, showing that respondents’ gender (*p* = 0.01), occupation (*p* = 0.03), and place of residence (either an NNOA or NOA) (*p* = 0.04) were the significant factors impacting the knowledge and perceptions of bat ecology and myths. 

The output of the adjusted logistic regression analysis is provided in Table 4. The results showed that the respondents who were farmers had 0.34 times the odds of having a positive or correct perception of bat ecology and myths about bats (OR = 2.99, 95% CI = 1.15–7.81, *p* = 0.03) compared to the respondents who were businesspersons. There was no significant variation found for the other variables. 

### 3.3. Knowledge, Perceptions, and Attitudes on the Spreading of NiV by Area

Nine statements asked the respondents to assess their knowledge, attitudes, and perceptions of the spreading of NiV by bats (Table 5). Overall, the results under this theme indicated that people have incorrect knowledge of the spreading of NiV. Specifically, more than half of the respondents (*n* = 117) disagreed (SD + D) with the statement “All bats carry Nipah virus”. This response was found to be higher in NOAs, and the difference was statistically significant (*p* = 0.01). On the contrary, most of the respondents (*n* = 113) agreed with the statement “Only bats are responsible for NiV outbreaks in Bangladesh” with a significant difference (*p* = 0.00), and this response was higher in NNOAs.

While asking whether “Even the presence of bats in my house can spread NiV”, the results showed an interesting pattern. Almost half of the respondents from NNOAs agreed (SA + A), while the other half disagreed (SD + D); meanwhile, in the NOAs, nearly two thirds (*n* = 62) of respondents agreed (SA + A) and the rest disagreed. This difference was statistically significant (*p* = 0.00). The majority of the respondents disagreed (*n* = 133) with the statement “I eat bat-bitten fruit without washing the fruit”, and this response was found to be higher in NNOAs (*n* = 77), with a statistically significant difference (*p* = 0.00). Most of the respondents (*n* = 152) agreed (SA + A) with the statement “I can identify fruits that have been bitten by bats”, showing correct responses with a significant difference between NNOAs and NOAs (*p* = 0.00).

Similarly, most of the respondents (*n* = 112) disagreed with the statement “Bats spread Nipah by biting humans and animals”. An area-wise comparison showed that respondents from NOAs were more likely to disagree (*n* = 70) compared to those from NNOAs (*n* = 42). In contrast, respondents from NNOAs were more likely to agree (*n* = 67) than those from NOAs (*n* = 29), demonstrating incorrect knowledge among NNOA respondents. Moreover, the responses were statistically significant (*p* = 0.00). The majority of the respondents (*n* = 120) disagreed (SD + D) with the statement “Walking under the roosting site can lead to NiV infection”, with a similar trend of responses between NNOA and NOA respondents, indicating that the respondents from both NOAs and NNOAs had incorrect knowledge of this item.

In total, 60 out of 109 respondents in NNOAs and 54 out of 99 in NOAs agreed (SA + A) that “Bats can only spread Nipah through date palm sap”, which reflects incorrect knowledge of this item. The difference between agreement and disagreement among respondents from both areas was statistically significant (*p* = 0.03). However, most of the respondents (*n* = 112) agreed (SA + A) with the statement “Drinking boiled date palm sap once boiled can reduce the risk of NiV infection”, showing a statistically significant difference (*p* = 0.00). An area-wise comparison showed that respondents from NNOAs were more likely to disagree (*n* = 64) with this statement compared to those from NOAs (*n* = 32).

#### Differences in Respondents’ Knowledge, Perceptions, and Attitudes on the Spreading of NiV

Principle factor analysis was performed to show the underlying significant (*p*-values < 0.05) factors between demographic variables and the spreading of NiV in NNOAs and NOAs. The results are shown in Table 6. Age, occupation, and level of education were found to be significant factors impacting the knowledge of the spreading of NiV in NNOAs (*p* = 0.00, *p* = 0.00, and *p* = 0.00, respectively). On the other hand, in NOAs, occupation and level of education were found to be significant factors of the same theme (*p* = 0.00 and *p* = 0.01, respectively). 

The adjusted regression analysis results showed that the respondents with a service occupation had 3.02 times the odds (OR = 3.02, 95% CI = 1.07–8.54, *p* = 0.04) of having correct knowledge on the spreading of NiV compared to businesspersons (Table 7). Moreover, the respondents who had completed the primary level of education were 3.06 times (OR = 3.06, 95% CI = 1.02–9.17, *p* < 0.05) more likely to have correct knowledge compared to those that had completed the higher education level.

## 4. Discussion

We often observed that the perceptions of the community people were not logical or scientific, although in a few instances, they were on the right track. In this discussion, we would like to correlate the questionnaire responses with a scientific and conservation perspective, thus making it possible to conclude the strength of knowledge and awareness among the community people. The public perception of bats has historically been mostly negative, with bats often portrayed as birds or dirty animals [33]. Despite having an important role in the ecosystem, bats are considered a fearful and mysterious animal by the community people [34]. In this study, we also found similar results. People had incorrect knowledge of bat ecology. The analysis revealed that a significant number of respondents agreed or strongly agreed with some negative statements (e.g., “Bats are birds”, “Bats are dirty animals”, and “Bats are blind”). Bats play an essential role in the ecosystem in terms of pest control and plant pollination. Despite the benefits of bats to the environment and the economy, bats are suffering because of the different myths about them believed by humans. The results for the different statements about the myths of bats indicate negative perceptions based on incorrect information about bats. In particular, the responses to “Bat meat and bones can cure different diseases” might be responsible for the medical value placed on bat organs in curing different deadly diseases. This finding is aligned with previous findings from a study that was conducted in Bangladesh [35]. Such a belief may motivate people to consume or use bat meat or other organs. In many studies [36,37,38], it was found that humans consume bat meat as part of their traditional and cultural beliefs.

Bats are victims of turbines, human encroachment, pesticides, and disease conditions [39]. Because of their critical importance to the environment, humans should do what they can to protect bats [33]. Along with economics, bats also have ecological value as seed dispersers of different plants, thus being essential pollinators. Globally, bats are involved in the pollination of approximately 528 species of plants of different families, such as Agavaceae and Cactaceae, which rely significantly on bats [40]. Bats assist in the dispersal of seeds of plant species, such as figs and palms, by ingesting and then carrying them to new locations [41]. In these cases, the seeds germinate in the digestive tract of bats, and they often travel a substantial distance before expelling the seeds, allowing them to take root and grow in new settings. 

Additionally, bats have been reported to move pollen from between 800 m and 18 km away from the home tree [42]. By supporting the pollination and dispersal of seeds, bats play an essential role in preserving the biodiversity of ecosystems. In the current study, most of the respondents agreed or strongly agreed that bats are important for nature; however, they also showed reluctance in supporting bat protection.

Although the Chiropteran order has been shown to provide significant benefits to humans, both economically and environmentally, bats are under threat, with the numbers of some species experiencing radical decreases. According to the International Union for Conservation of Nature and Natural Resources (IUCN), the known bat species are categorized as being of least concern for extinction. Threats to bats that may influence bat populations include disease, habitat destruction, construction of wind turbines, urbanization in the habitat area, and the negative social image of bats. The bat population is decreasing because of the displacing force resulting from the increasing invasion of their environments by human beings. With the increasing habitat destruction due to urbanization, bats are increasingly finding homes in human habitats, leading to human–bat conflict. This displacement of bats leads to lower reproductive success and increased mortality [43]. Bats are linked to many fatal disease outbreaks in humans, such as SARS-CoV and the Ebola virus [44,45]. It is also popularly believed that bats are responsible for NiV outbreaks in Bangladesh, and a similar trend was observed in this study. Although the value for Cronbach’s alpha was not satisfactory for indicating reliability in the analysis on NiV transmission in the current study, we performed principle factor analysis and chi-square tests to identify significant associations with respondents’ demographics. The findings regarding the knowledge of NiV spread revealed that respondents from NNOAs were more likely to believe that bats are responsible for the transmission of NiV to humans and that all bats carry NiV. Among the respondents in all areas, knowledge of behavioral aspects and how bats spread NiV was also found to be incorrect. For example, most of the respondents from both areas disagreed or strongly disagreed that walking under the roosting site can lead to NiV infection. Similarly, it was also widely believed that bats could only transmit NiV through consumption of raw date palm sap, considered to be one of the most significant routes of NiV transmission [46]. However, risky human behaviors, such as coming into close contact with an infected person, are also responsible for NiV outbreaks [47].

Studies on human–bat interactions suggest that the transmission of diseases is at least somewhat due to animal–human conflict. For instance, in 2007, the Ebola hemorrhagic fever outbreak in the Democratic Republic of the Congo resulted from hunting of migrating fruit bats for bushmeat by villagers [48]. Subsequently, other factors such as modern transportation and traditional cultural practices, where family members must maintain contact with sick persons, may contribute to the spread of such diseases [49]. However, creating a conservation education program to connect people with nature is not easy [50,51]. If the public is going to put forth an effort to help save bat populations, then they must adopt a more positive view on bats. As mentioned, this can be best accomplished by educating community people about bat ecology and conservation. Various incorrectly held public thoughts and beliefs, such as that bats do nothing but harbor diseases and that they are vampires or are related to witchcraft, a symbol of danger, etc., have placed bats in opposition to human beings, leading to a potential threat to the survival of bats. Nowadays, education programs are being launched in several countries, including an increase in bat population studies in the United States [33,52,53] and the United Kingdom [54], which show that community education can improve the perceptions of bats held by community people in a more positive direction. There is a clear reflection observed in our study in terms of agreement with positive statements and disagreement with incorrect statements in NOAs. Additionally, college students who were more knowledgeable about bats also had a more positive view of bats [55]. Conversely, in our study, we found that level of education was strongly inversely associated with holding myths about bats and having incorrect knowledge of the spreading of NiV. However, due to the nature of the study design, the current study is unable to explain why these factors are important predictors. Therefore, we strongly recommend further study that is preferably qualitatively exploratory in nature in order to provide explanations from social and cultural perspectives. For preventive measures of NiV spillover, awareness programs should be implemented, including for mitigation strategies, such as the use of bamboo skirts during the collection of date palm sap as well as the avoidance of drinking raw date palm sap and eating half-eaten fruits. Moreover, community-based educational campaigns should be organized in high-risk bat–human interface areas targeted at bat hunters and consumers and Gachi (tree nursers) as well as the households neighboring bat roosts in order to avoid future outbreaks of NiV in Bangladesh.

## 5. Conclusions

The findings of the current study allowed us to understand and identify the misconceptions and misunderstandings regarding bats. This study provides some valuable insights into the perceptions of bat ecology, the myths associated with bats, and the knowledge of the spreading of NiV held by community people. Methodologically, this study assessed people’s knowledge, perceptions, and attitudes toward bats in both NNOAs and NOAs. The age variable was statistically associated with knowledge of bat ecology, myths about bats, and perception of the spreading of NiV. By contrast, the outbreak status of an area was significantly related to myths about bats. The level of education had an association with myths about bats and knowledge of the spreading of NiV. Considering the results, this study strongly recommends designing an appropriate intervention program targeting community people in order to change their negative perceptions of bats and their incorrect knowledge regarding NiV transmission. People from different age categories, occupations, levels of education, and gender should be targeted when designing such an educational intervention.

## Figures and Tables

**Table 1:** The demographic characteristics of study participants.

Variables	NNOAs *n* (%)	NOAs *n* (%)
Sex		
Male	91 (83.5)	93 (94)
Female	18 (16.5)	6 (6.0)
Age		
≤24	15 (13.8)	22 (22.22)
25–34	44 (40.3)	37 (37.3)
35–44	35 (32.1)	23 (23.2)
45–54	13 (11.9)	15 (15.1)
≥55	2 (1.8)	1 (1.48)
Occupation		
Business	14 (12.8)	33 (33.3)
Farmer	50 (45.8)	39 (39.4)
Service	18 (16.5)	16 (16.1)
Student	26 (23.8)	10 (10.1)
Other	1 (0.9)	1 (1.0)
Education		
Higher	27 (24.7)	11 (11.1)
Primary	53 (48.6)	44 (44.4)
Secondary	29 (26.6)	44 (44.4)

NOAs: Nipah outbreak areas; NNOAs: Nipah non-outbreak areas.

**Table 2:** Knowledge, attitudes, and perceptions of the ecology and myths of bats.

Items	Strongly Disagree *n* (%)	Disagree *n* (%)	Agree *n* (%)	Strongly Agree *n* (%)
Bats are birds *	70 (33.7)	99 (47.6)	6 (2.9)	33 (15.8)
Bats are dirty animals *	7 (3.3)	69 (33.2)	90 (43.3)	42 (20.2)
Bats are blind *	24 (11.5)	72 (34.6)	66 (31.7)	46 (22.1)
Bats lay eggs *	51 (24.52)	115 (55.3)	24 (11.5)	18 (8.6)
Bats are look like foxes	44 (21.1)	93 (44.7)	61 (29.2)	10 (4.8)
Bats eat and defecate in the same place (i.e., the mouth) *	14 (6.7)	45 (21.6)	63 (30.3)	86 (41.4)
Bat meat and bones can cure different diseases *	12 (5.8)	89 (42.8)	79 (37.9)	28 (13.5)
Bats can get entangled in hair *	30 (14.4)	82 (39.4)	25 (2.4)	91 (43.8)
Bats are important in nature (e.g., for pollination, seed dispersal, and insect control)	9 (4.3)	62 (29.8)	81 (38.9)	56 (26.9)
I am not interested about whether bats in Bangladesh are endangered *	16 (7.6)	62 (29.8)	107 (51.4)	23 (11.1)
Bat populations are decreasing day by day	6 (2.9)	49 (23.5)	107 (51.4)	46 (22.1)
Greater attention should be provided for bat protection	19 (9.1)	83 (39.9)	83 (39.9)	23 (11.1)
Bats always attack human eyes *	20 (9.62)	134 (64.4)	36 (17.3)	18 (8.7)
All bats suck blood from humans *	34 (16.4)	136 (65.4)	24 (11.5)	14 (6.7)
Bats are a sign of bad things *	22 (10.6)	95 (45.7)	72 (34.6)	19 (9.1)
I like to read books about bats	30 (14.4)	102 (49.0)	52 (25.0)	24 (11.5)

* Negative/Incorrect statement.

**Table 3:** Test of statistical significance of variation in the respondents’ knowledge, attitudes, and perceptions of the ecology and myths of bats by their characteristics.

Variables		Negative *n* (%)	Positive *n* (%)	*p*
Age	≤24	21 (20.0)	16 (15.5)	0.153
	25–34	36 (34.3)	45 (43.7)	
	35–44	35 (33.3)	23 (22.3	
	45 or more	13 (12.4)	19 (18.6)	
Gender	Female	18 (17.1)	6 (5.8)	0.011
	Male	87 (82.9)	97 (94.2)	
The area by outbreak status	NNOAs	63 (60.0)	46 (44.7)	0.027
	NOAs	42 (40.0)	57 (55.3)	
Occupation	Businessperson	15 (14.3)	32 (31.1)	0.044
	Farmer	52 (49.5)	37 (35.9)	
	Service	16 (15.2)	18 (17.5)	
	Student	21 (20.0)	15 (14.6)	
	Other	1 (1.0)	1 (1.0)	
Education	Higher	23 (21.9)	15 (14.6)	0.106
	Primary	52 (49.5)	45 (43.7)	
	Secondary	30 (28.6)	43 (41.8)	

**Table 4:** Logistic regression analysis of the factors associated with respondents’ knowledge, attitudes, and perceptions of the ecology and myths of bats.

Variables		OR, 95% CI, *p*
Age	≤24	Ref
	25–34	2.42, 0.94–6.26, 0.068
	35–44	1.15, 0.41–3.24, 0.784
	45 or more	3.05, 0.95–9.83, 0.062
Gender	Female	Ref
	Male	2.95, 1.00–8.66, 0.050
The area by outbreak status	NNOAs	Ref
	NOAs	1.42, 0.76–2.63, 0.269
Occupation	Businessperson	Ref
	Farmer	0.34, 0.15–0.78, 0.011
	Service	0.57, 0.20–1.61, 0.287
	Student	0.70, 0.21–2.33, 0.562
	Other	0.91, 0.05–16.94, 0.952
Education	Higher	Ref
	Primary	1.44, 0.50–4.17, 0.503
	Secondary	1.99, 0.77–5.14, 0.154
OR: odds ratio; 95% CI: 95% confidence interval.		

**Table 5:** Knowledge, attitudes, and perceptions of the spreading of NiV by bats.

Items	NNOAs (*n* = 109)				NOAs (*n* = 99)				*p*
	Strongly Disagree *n* (%)	Disagree *n* (%)	Agree *n* (%)	Strongly Agree *n* (%)	Strongly Disagree *n* (%)	Disagree *n* (%)	Agree *n* (%)	Strongly Agree *n* (%)	
All bats carry NiV *	8 (7.3)	48 (44.04)	44 (40.4)	9 (8.26)	4 (4.0)	57 (57.6)	22 (22.2)	16 (16.1)	0.012
Only bats are responsible for Nipah outbreaks in Bangladesh *	0	41 (37.6)	54 (49.5)	14 (12.8)	4 (4.0)	50 (50.5)	27 (27.2)	18 (18.2)	0.003
Even the presence of bats in my house can spread NiV *	2 (1.8)	50 (45.9)	47 (43.1)	10 (9.1)	4 (4.0)	58 (58.6)	19 (19.2)	18 (18.2)	0.002
I eat bat-bitten fruit without washing the fruit *	44 (40.3)	33 (30.3)	22 (20.1)	10 (9.17)	18 (18.1)	39 (39.3)	16 (16.1)	26 (26.2)	0.000
I can identify fruits that have been bitten by bats	10 (9.1)	6 (5.5)	62 (56.9)	31 (28.4)	14 (14.1)	26 (26.2)	26 (26.2)	33 (33.3)	0.000
Bats spread NiV by biting humans and animals *	2 (1.8)	40 (36.7)	56 (51.3)	11 (10.0)	8 (8.8)	62 (62.6)	20 (2.2)	9 (9.0)	0.000
Walking under the roosting site can lead to NiV infection	4 (3.7)	54 (49.5)	45 (41.3)	6 (5.5)	10 (10.1)	52 (52.5)	33 (33.3)	4 (4.0)	0.223
Bats can only spread NiV through date palm sap *	2 (1.83)	45 (41.3)	48 (44.0)	14 (12.8)	12 (12.1)	33 (33.3)	42 (42.4)	12 (12.1)	0.028
Drinking boiled date palm sap once boiled can reduce the risk of NiV infection	6 (5.5)	58 (53.2)	26 (23.9)	19 (17.4)	12 (12.12)	20 (20.2)	42 (42.4)	25 (25.2)	0.000

* Negative/Incorrect statement.

**Table 6:** Test of statistical significance of the variation in the respondents’ knowledge, attitudes, and perceptions of the spreading of NiV by their characteristics.

Variables		NNOAs (*n* = 109)			NOAs (*n* = 99)		
		Negative	Positive	*p*	Negative	Positive	*p*
Age	≤24	14 (27.5)	1 (1.7)	0.001	13 (24.5)	9 (19.6)	0.093
	25–34	21 (41.2)	23 (39.7)		14 (26.4)	23 (50.0)	
	35–44	11 (21.5)	24 (41.3)		14 (26.4)	9 (19.6)	
	45 or more	5 (9.8)	15 (13.7)		12 (22.6)	5 (10.8)	
Gender	Female	10 (19.6)	8 (13.8)	0.415	1 (1.9)	5 (10.9)	0.062
	Male	41 (80.4)	50 (86.2)		52 (98.1)	41 (89.1)	
Occupation	Businessperson	9 (17.6)	5 (8.6)	0.000	18 (33.9)	15 (32.6)	0.002
	Farmer	9 (17.6)	41 (70.7)		28 (52.8)	11 (23.9)	
	Service	11 (21.6)	7 (12.0)		2 (3.8)	14 (30.4)	
	Student	22 (43.1)	4 (6.9)		5 (9.4)	5 (10.8)	
	Other	-	1 (1.7)		-	1 (2.1)	
Education	Higher	20 (39.2)	7 (12.0)	0.000	4 (7.5)	7 (15.2)	0.012
	Primary	13 (25.5)	40 (69.0)		25 (47.2)	19 (41.3)	
	Secondary	18 (35.3)	11 (19.0)		24 (45.3)	20 (43.5)	

**Table 7:** Logistic regression analysis of the factors associated with respondents’ knowledge, attitudes, and perceptions of the spreading of NiV.

Variables		OR, 95% CI, *p*
Age	≤24	Ref
	25–34	2.32, 0.87–6.22, 0.093
	35–44	2.03, 0.70–5.91, 0.190
	45 or more	2.38, 0.33–3.49, 0.914
Gender	Female	Ref
	Male	0.57, 0.20–1.62, 0.293
The area by outbreak status	NNOAs	Ref
	NOAs	1.02, 0.43–2.42, 0.966
Occupation	Businessperson	Ref
	Farmer	1.56, 0.70–3.47, 0.272
	Service	3.02, 1.07–8.54, 0.037
	Student	0.70, 0.20–2.44, 0.576
	Other	1, -, -
Education	Higher	Ref
	Primary	3.06, 1.02–9.17, 0.045
	Secondary	1.90, 0.71–5.12, 0.138

## Supplementary Materials

The following are available online at https://www.mdpi.com/2076-2615/10/10/1814/s1, Table S1: Survey data of understanding the community perceptions and knowledge of bats and Transmission of Nipah Virus in Bangladesh.
